# Detection and Management of Mango Dieback Disease in the United Arab Emirates

**DOI:** 10.3390/ijms18102086

**Published:** 2017-10-20

**Authors:** Esam Eldin Saeed, Arjun Sham, Ayah AbuZarqa, Khawla A. Al Shurafa, Tahra S. Al Naqbi, Rabah Iratni, Khaled El-Tarabily, Synan F. AbuQamar

**Affiliations:** 1 Department of Biology, United Arab Emirates University, Al-Ain 15551, UAE; esameldin_saeed@uaeu.ac.ae (E.E.S.); arjunsham@uaeu.ac.ae (A.S.); 201250620@uaeu.ac.ae (A.A.); R_Iratni@uaeu.ac.ae (R.I.); 2 Ministry of Climate Change and Environment, Sharjah 1509, UAE; kaalshurafa@moccae.gov.ae (K.A.A.S.); tsalnaqbi@moccae.gov.ae (T.S.A.N.)

**Keywords:** dieback, disease management, *Lasiodiplodia theobromae*, mango, pathogenicity

## Abstract

Mango is affected by different decline disorders causing significant losses to mango growers. In the United Arab Emirates (UAE), the pathogen was isolated from all tissues sampled from diseased trees affected by *Lasiodiplodia theobromae*. Symptoms at early stages of the disease included general wilting appearance of mango trees, and dieback of twigs. In advanced stages, the disease symptoms were also characterized by the curling and drying of leaves, leading to complete defoliation of the tree and discolouration of vascular regions of the stems and branches. To substantially reduce the devastating impact of dieback disease on mango, the fungus was first identified based on its morphological and cultural characteristics. Target regions of 5.8S rRNA (*ITS*) and elongation factor 1-α (*EF1-α*) genes of the pathogen were amplified and sequenced. We also found that the systemic chemical fungicides, Score^®^, Cidely^®^ Top, and Penthiopyrad^®^, significantly inhibited the mycelial growth of *L. theobromae* both in vitro and in the greenhouse. Cidely^®^ Top proved to be a highly effective fungicide against *L. theobromae* dieback disease also under field conditions. Altogether, the morphology of the fruiting structures, molecular identification and pathogenicity tests confirm that the causal agent of the mango dieback disease in the UAE is *L. theobromae*.

## 1. Introduction

Mango (*Mangifera indica* L.) is an evergreen fruit tree that is adapted to tropical and subtropical conditions. Mango cultivars vary considerably in fruit size, colour, shape, flavor, texture, and taste [1], and is cultivated in many regions of the world, including India, China, Pakistan, Mexico, Brazil, Egypt, and Nigeria [2]. In addition, mango production has increased in non-traditional mango producing areas including the UAE. According to the FAO (2014), UAE has significantly increased the cultivated area and the number of trees of mango (FAOSTAT; Available online http://faostat.fao.org/site/339/default.aspx), and growers have widely cultivated this crop due to its nutritional and economical values, and their delicacy in flavour and taste. Recently, mango has become an increasingly popular fruit in the UAE markets, after dates and citrus. Mango suffers from diseases worldwide caused by a variety of pathogens that affect all parts of the tree and, therefore, reduce yield and quality of the fruit [3,4,5].

Mango decline or dieback is a serious disease of mango. The causal agent of this disease remained uncertain for many years due to different fungi associated with it [4]. Fungal pathogens, such as *Neofusicoccum ribis*, *Botryosphaeria dothidea*, *Diplodia* sp., *Pseudofusicoccum* sp., and *Ceratocystis* sp. may infect mango trees individually, or in combinations, to cause mango dieback in different parts of the world [5,6,7,8,9,10]. Botryosphaeriaceae species, such as *Lasiodiplodia hormozganensis*, *L. iraniensis*, and *L. egyptiacae* have also been associated with mango dieback in Iran, Australia, and Egypt [10,11,12]. *Lasiodiplodia theobromae* (Syn: *Botryodiplodia theobromae*) [13,14], however, it has been reported as the causal agent in destroying mango orchards within days or a few weeks of infection in India, USA, Pakistan, Brazil, Oman, and Korea [15,16,17,18,19,20]. *L. theobromae* is a soil-borne wound pathogen that can affect all parts of the mango tree at all ages. Consequently, mango dieback is considered to be an important problem confronting the mango industry and marketing [21]. To date, the mango dieback disease nor its causal organism has been reported from the UAE.

The fungus, *L. theobromae*, often invades twigs and branches from their tips of mango trees causing them to dry and the plant to wilt [22]. Under favourable conditions, infections are characterized by dying back of twigs from the top, downwards, followed by discolouration and the death of leaves, particularly in older trees, which gives an appearance of fire scorch. Symptoms can also be observed on reproductive structures [23]. In severe situations, branches start drying one after another in a sequence resulting in death of the trees of the mango plantation. Commonly, once the symptoms of decline or widespread dieback are evident, it is difficult to stop or reverse the progress of disease. The disease has also been observed on different mango varieties associated with the variation in their susceptibility towards the fungus. Reports have shown that certain varieties are highly susceptible [24,25]. In vivo studies demonstrated that *L. theobromae* becomes aggressive in colonizing host tissues when plants are under abiotic stress, such as heat, water stress, or drought stresses [26,27]. In general, dieback is one of the deadly diseases of mango, which causes a serious damage to the tree and its productivity.

To manage dieback disease, traditional horticultural practices have been applied to confront the fungal attack. In general, avoidance of wounding of trees can limit disease incidence [28]. Infected parts should be pruned from 7–10 cm below the infection site, removed, and burnt [29]. Attempts to arrest early infections have been made by treating with copper oxychloride or pasting with cow dung on pruned ends [30]. Biological control (e.g., *Trichoderma* spp.) have also been tried to reduce disease incidence of *L. theobromae* under in vitro and in field conditions [31,32]. Implementation of integrated disease management (IDM) programs which combine cultural, chemical, and biological approaches are highly recommended to control mango dieback, reduce cost, and improve production efficiency. Despite its negative impact on the environment and human health, the use of chemicals continues to be the major strategy to lessen the menace of crop diseases. In this study, we report fungicide treatments against *L. theobromae* as an effective and reliable approach to reduce the economic losses associated with mango dieback disease. Growers in the UAE and other mango producing countries experiencing this damaging disease are expected to directly benefit from the outcome of this study. Future physiological and molecular analyses will shed more light on dieback disease and its causal agent, which will ultimately lead to the development of effective IDM strategies to manage this disease. Here, we aimed not only to determine the etiology of this disease on mango trees in the UAE, but also to evaluate some of the available fungicides for their effect on the pathogen under in vitro and in vivo conditions.

## 2. Results

### 2.1. Symptoms of Dieback Disease on Mango

**Figure 1:**
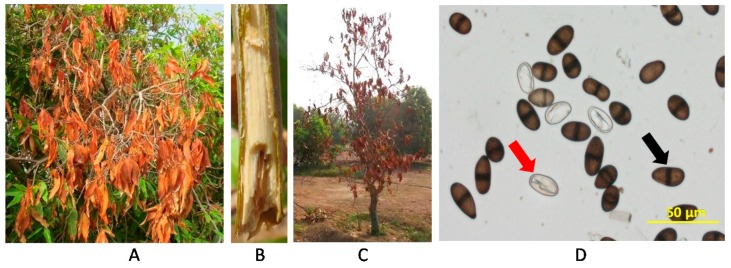
Naturally-infested mango trees showing symptoms of dieback disease and morphological phenotypes of *Lasiodiplodia theobromae* conidia. Symptoms on (**A**) leaves; (**B**) twigs; (**C**) whole tree; and (**D**) *L. theobromae* hyaline, aseptate immature (red arrow) and brown, 1-septate, thick-walled mature conidia (black arrow) from a 10-day old potato dextrose agar (PDA) culture.

Trees manifested with disease symptoms from Kuwaitat, Al Ain—in the eastern region of Abu Dhabi Emirate, UAE—were reported. The pathogen was observed to attack different parts of the mango trees. First, we noticed the disease symptoms in all plant tissues, including leaves, twigs, and apical tips. When the fungus attacks the leaves, their margins roll upwards (Figure S1) turning them a brownish colour (Figure 1A). Later, a scorch-like appearance developed, followed by the dropping of the infected leaves. Moreover, twigs died from the tips back inwards (toward the vascular tissues) (Figure 1A), giving a scorched appearance to the branches (Figure S1). We observed browning in the vascular tissues when longitudinal cross-sections were made in diseased mango twigs (Figure 1B). We also determined the disease symptoms associated with dieback on whole trees in the field.

At later stages of invasion, disease symptoms such as wilting, complete drying of leaves and death of the apical region of plants, may also appear (Figure 1C) and at different ages of mango trees (Figure S1). In general, branches dry out one after another in a sequence resulting in the eventual death of the whole tree. These symptoms on mango are typical of the dieback disease.

### 2.2. Morphological and Phylogenetic Identification of L. theobromae Associated with Dieback Disease

The isolate obtained on potato dextrose agar (PDA) and sporulation from naturally-affected tissues associated with dieback disease on mango trees (Figure 1A–C) were microscopically examined. On PDA, colonies of *L. theobromae* (Pat.) Griffon and Maubl. [13,14] had initial white aerial mycelia that turned greenish-gray mycelium with age (Figure S1). The mycelium produced dark brown to black conidia. We also observed mycelial growth and production of immature and mature conidia (Figure 1D). Immature conidia were subovoid or ellipsoid, thick-walled, hyaline and one-celled, turning dark brown, two-celled and with irregular longitudinal striations when at maturity. The size of mature conidia averaged 26.6 ± 0.51 μm long and 12.9 ± 0.28 μm wide. This suggests that *L. theobromae* is most likely the causal organism of dieback in mango.

**Figure 2:**
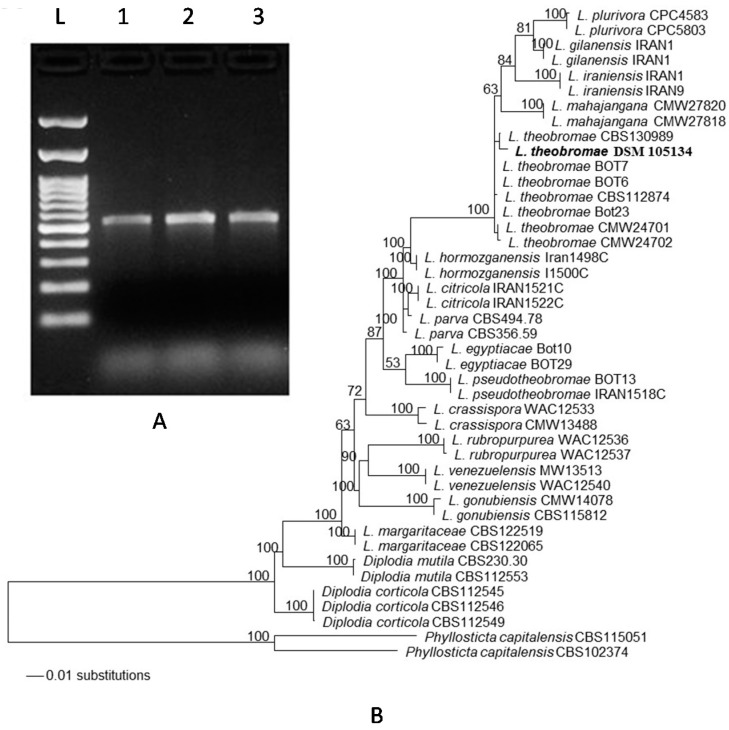
Molecular identification of *L. theobromae.* (**A**) PCR amplification of the *ITS* rDNA region in infected leaves, twigs and apical tips (lanes 1–3, respectively); and (**B**) dendrogram showing phylogenetic relationships of the fungal sequence of the specimen used in this study (DSM 105134) with the most related *ITS* and *TEF1-α* sequences in GenBank (accession number, MF114110), prepared by the neighbour-joining method. The maximum likelihood tree is obtained from combined *ITS*/*TEF1-α* sequence data. Numbers at the nodes are ML bootstrap values after 100 replicates are expressed as percentages (LnL = −3497.793130). The scale bar on the rooted tree indicates a 0.01 substitution per nucleotide position. The strain from this report is indicated in bold. *ITS*, internal transcribed spacer; *TEF1-α*, translational elongation factor *1-α*; L, DNA ladder.

We also established a phylogenic analysis of the isolate. PCR amplification of internally-transcribed spacers (*ITS*) of the rDNA gene from mycelium of infected tissues subcultured on PDA was carried (Figure 1). Our results detected the *ITS* gene of all infected tissues (Figure 2A), confirming that *L. theobromae* is frequently associated with all dieback disease symptoms on mango trees in the UAE. To check if the DNA sequences of this species collected in the UAE belongs to any isolated *Lasiodiplodia* isolate, we compared the identified strain with those available in GenBank based on a phylogeny tree. For that purpose, the *ITS* rDNA and the translational elongation factor 1-α (*TEF1-α*) gene [33] were used as a single gene set. The concatenated two-gene set (*ITS* and *TEF1-α*) were sequenced and deposited in GenBank (accession number: MF114110 and MF097964, respectively).

We also determined the relationship among this obtained and other closely related *ITS*/*TEF1-α* sequences [12,30]. All sequences were aligned and maximum likelihood analyses were performed for estimation of the phylogenetic tree. The adaptation to different plant hosts has led to the evolution of at least 13 cryptic species within the *L*. *theobromae* species complex [12]. The generated *ITS*/*TEF1-α* sequence belonging to our strain clustered in one clade corresponding to *L. theobromae* from different sources, confirming its identity with this species (Figure 2B). Among the studied *Lasiodiplodia* species, our analysis revealed that this pathogen is placed adjacent to *L. theobromae* CBS130989, distinguishing the obtained isolate from those belonging to other species of *Lasiodiplodia*, *Diplodia*, or *Phyllosticta*. Our phylogenetic analysis supports that the species *L. theobromae* (collection number DSM 105134) dominates in the UAE causing dieback disease on mango trees.

### 2.3. Pathogenicity Tests of L. theobromae on Mango Leaves, Fruits, and Seedlings

**Figure 3:**
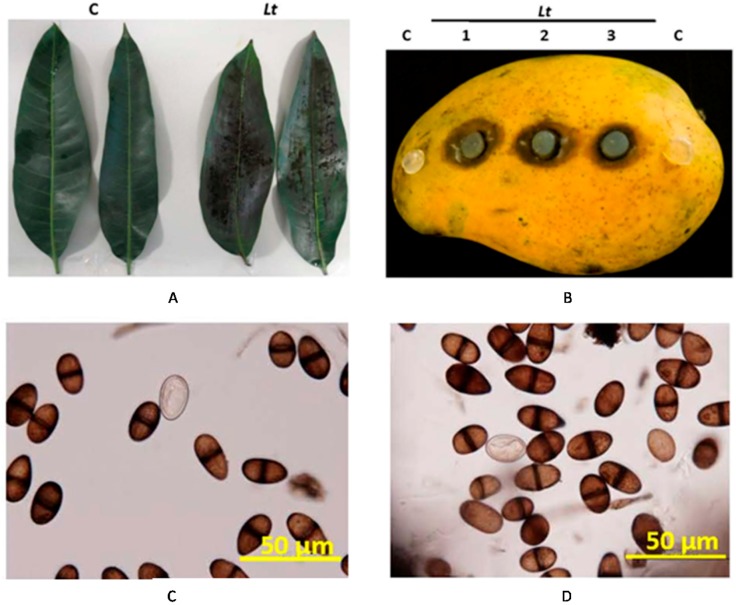
Pathogenicity assays of *L. theobromae* on mango leaves and fruits. Pathogenicity tests on inoculated (right) and non-inoculated (left) of (**A**) detached mango leaves; and (**B**) mango fruits, at 5 dpi. Conidia of the pathogen from the inoculated mango (**C**) leaves; and (**D**) fruits. C, control (no *L. theobromae*); *Lt*, *L. theobromae*.

To confirm our results, detached leaves were spray-inoculated with the isolated pathogen. Following inoculation, a black rot developed on the leaves after five days post-inoculation (dpi) (Figure 3A). No disease symptoms appeared on control leaves sprayed with sterilized distilled water. Similarly, we inoculated mango fruits with the same pathogen. On fruits, dark brown to black lesions averaged 26.4 mm in diameter, beneath the PDA plugs containing the pathogen were observed at 5 dpi (Figure 3B). No disease symptoms were evident under the control plug without the pathogen. The symptoms of the disease were evident on the inoculated leaves (Figure 3A) and fruits (Figure 3B), but not from the control tissues, fulfilling the Koch’s postulates relating to the pathogenicity of *L. theobromae* (Figure 3C,D). Our data suggest that *L. theobromae* causes the disease on different tested tissues of mango.

**Figure 4:**
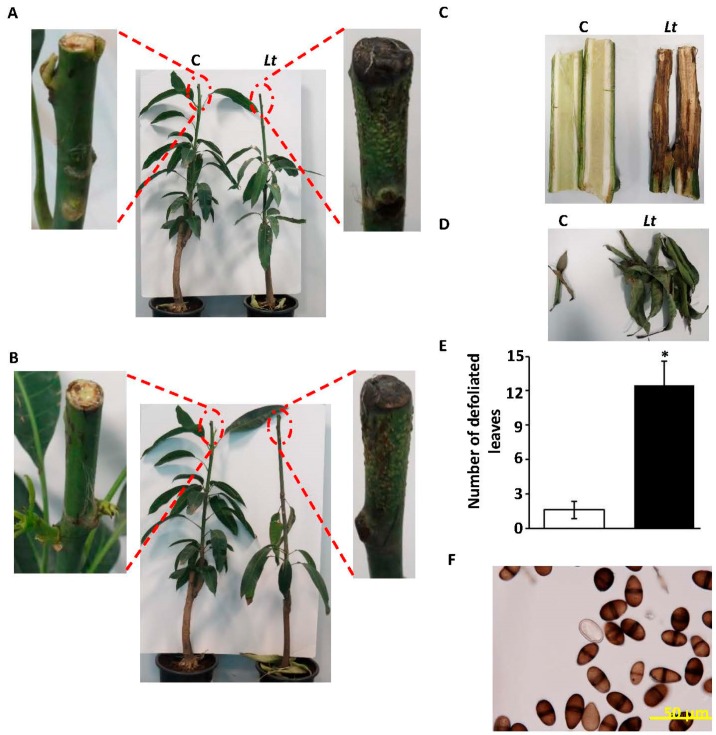
Pathogenicity and Koch’s postulate testing with *L. theobromae*. Pathogenicity test on inoculated (right) and non-inoculated (left) of seedlings at (**A**) 3 wpi; and (**B**) 5 wpi. Close-up views of symptomatic (right) and non-symptomatic (left) apical tip tissues at 3 and 5 wpi, respectively; (**C**) Longitudinal section of young stems showing browning of vascular tissues; (**D**) Defoliated leaves of inoculated (right) and control (left) seedlings; and (**E**) the number of defoliated leaves of inoculated (dark column) and control (clear column) seedlings, at 5 wpi. Asterisks are significantly different from the corresponding control at *p* < 0.05. (**F**) Conidia after re-isolation of the pathogen from colonized tissues.

In addition, we performed pathogenicity tests on healthy mango (cv Badami) seedlings, and monitored the disease progress. Plants were inoculated with 8 mm mycelial discs from 10-day old pure *L. theobromae* cultures grown in PDA, while control seedlings were inoculated with PDA without the pathogen. The seedlings were maintained under greenhouse conditions. Following inoculation, seedlings developed typical dieback symptoms showing a dark brown to black, necrotic tissues at the tip of the stem (point of inoculation). At the first week, black colour appeared on the stem at the site of inoculation (Figure S2). The disease progressed rapidly along the stem in the following weeks. At three weeks post-inoculation (wpi), symptoms often expressed as defoliated leaves and characterized by conidiomata development and tissue necrosis in inoculated plants (Figure 4A). At 5 wpi, seedlings showed complete black discolouration and necrosis of internal tissues of stems and branches (Figure 4B,C), forcing the leaves to fall (Figure 4D). Control leaf tissues remained symptomless. The pathogen was consistently re-isolated from the disease affected tissues; thus fulfilling Koch’s postulates that these detected symptoms were associated with the inoculation with the pathogen *L. theobromae* (Figure 4E).

### 2.4. In Vitro Evaluation of Systemic Fungicides Against L. theobromae

**Figure 5:**
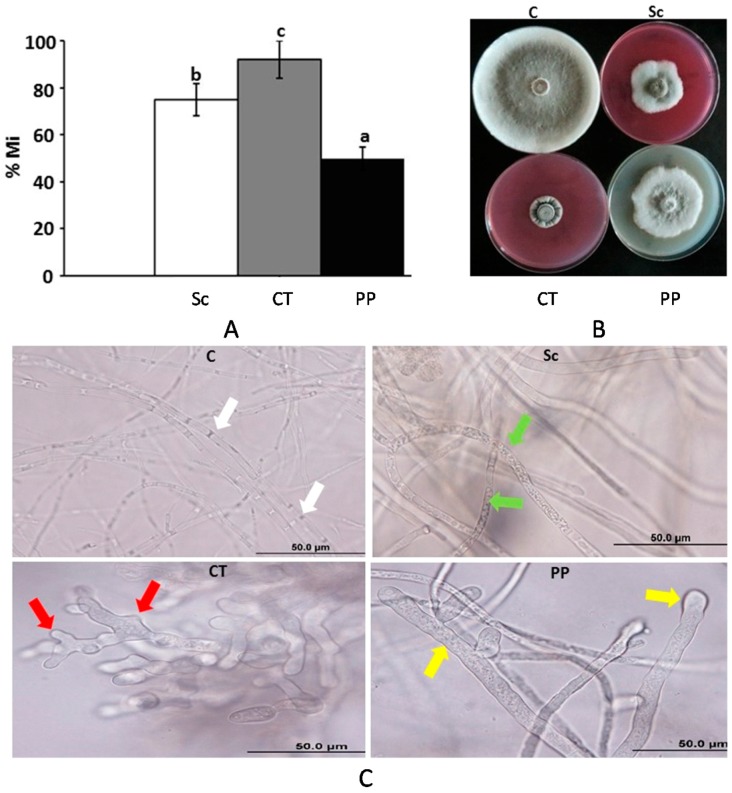
Efficacy of fungicides against *L. theobromae*. (**A**) Growth inhibition rate (% Mi) of *L. theobromae* using 250 ppm of the fungicides after 10 days. Values with different letters are significantly different from each other at *p* < 0.05; (**B**) the effect of fungicides (250 ppm) on in vitro mycelial growth; and (**C**) abnormalities in hyphal morphology, septum formation, and cytoplasmic contents of *L. theobromae*, following fungicide treatments, compared to control. White arrows indicate normal septate hyphal growth; green arrows indicate formation of non-septate hyphal formation and cytoplasmic coagulation; red arrows indicate hyphal swellings and branch deformation; yellow arrows indicate hyphal swellings and cytoplasmic coagulation. C, control (no fungicide); Sc, Score^®^; CT, Cidely^®^ Top; PP, Penthiopyrad^®^.

To evaluate the effect of fungicides, Score^®^ (difenoconazole), Cidely^®^ Top (difenoconazole and cyflufenamid), and Penthiopyrad^®^ (Carboxamide), on the mycelial growth of *L. theobromae*, six concentrations, ranging between 25 and 1000 ppm of selected fungicides were applied in vitro (Figure S3). With the exception of Cidely^®^ Top, there was significant difference among the concentrations of the other two tested fungicides below 250 ppm, in inhibiting the mycelial growth of the causal agent of dieback disease, *L. theobromae* (Figure S3). On the other hand, Cidely^®^ Top fungicide increased fungal inhibition zone even at low concentration i.e., 25 ppm, and showed no, or slightly, significant deference when compared with other concentrations, ranging between 76–98% mycelial growth inhibition (Figure 5A). We also compared mycelial growth inhibition of *L. theobromae* in vitro at 250 ppm, which was considered as the most efficient concentration in the three fungicides. The results indicated that Score^®^, Cidely^®^ Top, and Penthiopyrad^®^ inhibited the mycelial growth and sporulation of *L. theobromae* by 77%, 92%, and 50%, respectively (Figure 5A,B). This suggests that the systemic fungicide, Cidely^®^ Top, was the most effective fungicide at 250 ppm concentration among all tested fungicides; and that the fungal inhibition zones were observed, even when a low dosage of this fungicide was applied.

In addition, microscopic examination was performed to find out the mode of action of the fungicides in inhibiting the growth of this fungal pathogen. The observations revealed that the fungicides caused diverse morphological alternations on *L. theobromae*. In comparison to the hyphal growth of *L. theorbromae* without any treatment, Score^®^ was capable of causing septal malformations in the hyphal cells (Figure 5C). Striking morphological abnormalities were observed in cell cultures of *L. theobromae* treated with Cidely^®^ Top. The fungicide affected the growth of the pathogen causing significant cytoplasmic coagulation, shrivelled or misshaped mycelia. On the other hand, Penthiopyrad^®^ caused considerable thickening of hyphal tips and incomplete septal formation (Figure 5C). Altogether, the selected systemic fungicides inhibited the mycelial growth of *L. theobromae* by inducing morphological abnormalities of *L. theobromae*. Although many reports in literature have noted pronounced fungal growth inhibition with fungicides under in vitro conditions, many have failed to repeat these performances under greenhouse or field conditions [34].

### 2.5. Effect of Fungicides on Mango Plants Infected with L. theobromae

**Figure 6:**
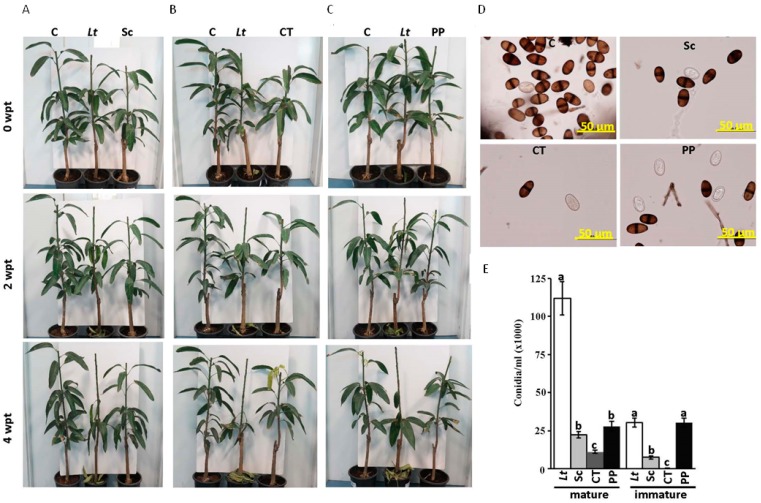
Effect of fungicide treatments on artificially inoculated mango seedlings with *L. theobromae* in the greenhouse. Fungicidal suppression of dieback disease on mango seedlings using (**A**) Score; (**B**) Cidely^®^ Top; (**C**) Penthiopyrad^®^ at 0 (top panel), 2 (middle panel), and 4 (bottom panel) wpt; (**D**) conidia of the pathogen reisolated from affected tissues of fungicide-treated plants; and (**E**) the number of conidia/mL at 4 wpt (6 wpi with *L. theobromae*). Seedlings inoculated with *L. theobromae* at two weeks before the fungicide treatment. Values with different letters are significantly different from each other at *p <* 0.05. C, control (non-inoculated seedling); *Lt*, *L. theobromae*-inoculated seedling; Sc, Score^®^; CT, Cidely^®^ Top; PP, Penthiopyrad^®^.

To confirm our results, we sprayed Score^®^, Cidely^®^ Top, or Penthiopyrad^®^ fungicides on diseased seedlings artificially inoculated with *L. theobromae* at 2 wpi, and measured the efficacy of the fungicide again after another four weeks (four weeks post treatment; wpt). Before the treatment with the fungicides (corresponding to 0 wpt), plants showed obvious dieback disease symptoms (Figure 6A–C). At 2 wpt with the fungicides, plants started to recover (Figure 6B) and prevented further disease progression at the assessment at 4 wpt (Figure 6A–C), which was in contrast to the plants sprayed with sterilized distilled water (*L. theobromae* control). We also observed the emergence of new leaves from the apical or auxiliary buds of seedlings treated with Score^®^ or Penthiopyrad^®^, that were comparable to untreated control samples (Figure S4). Cidely^®^ Top-treated disease affected seedlings not only recovered after 4 wpt (Figure 6B), but also showed vigorous vegetative growth (Figure S4). Since all fungicide-treated plants showed very limited disease symptoms with lesser leaf defoliation at 4 wpt, we decided to determine the effects of fungicides on conidia numbers and morphology. Although we did not notice any morphological malformation of the conidia obtained from plants treated with the fungicides, we expected a drop in the number of conidia produced (Figure 6D). Therefore, we counted the number of mature and immature conidia recovered from the tip of the stems of treated-mango plants. In general, there was a significant reduction in the number of mature conidia in all fungicide treatments (Figure 6E). The Cidely^®^ Top caused a greater reduction in the number of mature conidia, followed by Score^®^- and Penthiopyrad^®^-treated plants (Figure 6E). Although Score^®^ and Penthiopyrad^®^ had a similar reducing effect on the number of mature conidia, Score^®^ showed at least a three-fold reduction in immature conidia numbers of *L. theorbromae* when compared with Penthiopyrad^®^ fungicide. Application of Cidely^®^ Top resulted in the absence of the immature conidia. Our data suggest that *L. theobromae* appeared to lose some of its aggressiveness as a pathogen when the tested fungicides were applied; while a strong suppression was evident in the severity of the dieback disease in mango plants treated with Cidely^®^ Top.

**Figure 7:**
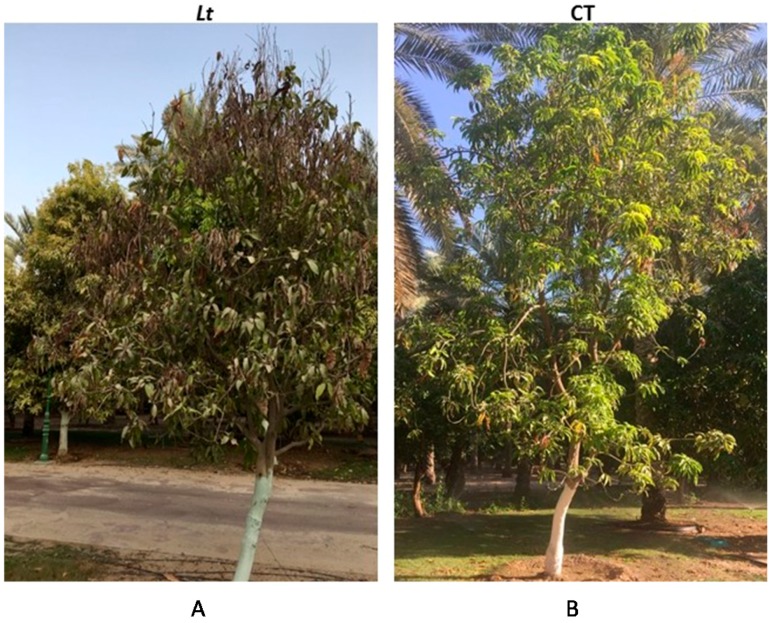
The effect of Cidely^®^ Top treatments on mango trees (cv Sindhri) naturally infected with *L. theobromae* in the field. Fungicidal suppression of dieback disease symptoms on mango trees (*n* = 12) non-treated (**A**) and treated with the fungicide Cidely^®^ Top (**B**) at 12 wpt. The photo on the left shows the condition of a diseased, affected tree; however, on the right, it shows another tree which was previously affected and has already recovered from severe disease symptoms.

In the field trials, the promising fungicide Cidely^®^ Top was applied to a mango orchard affected by dieback in order to confirm the results obtained from the in vitro and greenhouse experiments. Mango trees (cv Sindhri), were sprayed with 250 ppm of Cidely^®^ Top fungicide. The disease severity in the fungicide treated mango plants was gradually reduced already at 4 wpt of spraying with the fungicide (Table 1). It was also noted in the trees treated with Cidely^®^ Top that new vegetative growth comprising of fresh shoots increased after 12 wpt (Figure 7). As expected, the fungicide Cidely^®^ Top did not elicit any phytotoxic response on the cultivar under the field conditions. In untreated control plants, disease severity indices (DSI) increased with time in contrast to sprayed plants with the fungicide (Table 1). This suggests that the application with Cidely^®^ Top results in the complete disappearance of symptoms of the disease and the full recovery of the disease-affected trees.

## 3. Discussion

Mango (*Mangifera indica* L.) is known as “the king of fruits” because it is one of the most popular fruit of tropical regions [35]. The UAE has been motivated to widely grow mango in recent years [36]. *L. theobromae* [13,14] is a geographically widespread species of Botryosphaeriaceae [17,37], causing dieback disease in various mango growing areas in the world [9,10]. This fungal pathogen could be found alone or in combination with other fungal pathogens to cause dieback disease. Symptoms associated with this disease are expressed as twig tip dieback that advances into the old wood with branches that dry and die, and leaves scorch and fall, eventually causing death of plants. In the UAE, typical symptoms of dieback disease has been observed (Figure 1) and is yet to develop to an epidemic phase, causing fast-spreading death in mango orchards in a short period of time (i.e., about two months after the initial infection). Therefore, urgent need for appropriate and cost-effective research to properly manage this important disease. In this report, we aimed to determine the causal agent(s) of dieback on mango trees, and to find an effective solution for the potential threat associated with this disease in the UAE.

The pathogen was isolated and identified morphologically and phylogenetically. Microscopy demonstrated that the pathogen is a prolific producer of immature and mature conidia on PDA (Figure 1D). Consistent with Punithalingam [37], immature conidia were initially hyaline, unicellular, ellipsoid to oblong, thick walled with granular contents. We also observed that with age, mature conidia became two-celled, dark brown, with longitudinally striated appearance and an average size of 26.6 µm × 12.9 µm. On maturity, the size of conidia is about 20–30 µm × 10–15 µm [22,37]. In addition, the morphological characteristics of conidia were similar to those previously described [14]. The assessments of spore biology are important to distinguish the fungal survival, dispersal and pathogenicity among closely related species within *Botryosphaeriaceae* spp. [8,33], though we argue about the difficulty in identifying the species of the pathogen based merely on its conidial characteristics. To prove the microbial aetiology of the disease by verifying the existence of the pathogen and its progression in tissues, leaves, fruits and whole plants of mango were inoculated with the isolated pathogen (Figure 3 and Figure 4). The results of inoculation on tissues were similar to the disease symptoms in the field and the re-isolation of the pathogen from the inoculated plants confirmed Koch´s postulates. Our data match those in previous pathogenicity tests, which have been done on baobab (*Adansonia* sp.) [38], grapes (*Vitis vinifera*) [39], cocoa (*Theobroma cacao*) [40], yam (*Dioscorea alata*) [41], banana (*Musa* sp.) [42], and mango [12,22]. As in previous artificial inoculation trials on mango seedlings in Peru [43], our study, too, found that symptoms, such as blackening of shoot tips, partial death of crown areas, and defoliation of leaves, developed rapidly and were clearly evident after five weeks of inoculation. Thus, it would be virtually impossible to distinguish between *Lasiodiplodia* species based on their morphology only.

Specific genomic regions of *L. theobromae* that correspond to the two widely used loci *ITS* and *TEF1-α* were amplified and sequenced. Phylogenetic analysis of DNA sequences combining, *ITS* and *TEF1-α* [12,33,38], was also performed to discriminate between *Lasiodiplodia* species, and to identify the causal agent of the dieback disease on mango in the UAE. The adaptation to different plant hosts and environments has led to the evolution of at least 13 cryptic species within the *L*. *theobromae* species complex [12]. The identified *L*. *theobromae* DSM 105134 from the UAE fits into one clade with several *L*. *theobromae* strains from different sources. The most closely related *ITS/TEF1-α* was *L. theobromae* CBS 130989 (=BOT4), an isolate from mango in Egypt [12], which demonstrated an identity of 100%. Our data also showed that the *ITS/TEF1-α* identified in this study clustered together with *L. theobromae* isolates BOT 6, BOT 7, and BOT 23 from mango in Egypt [12]. The isolate CBS 112874 of *L. theobromae* was reported to infect grapes in South Africa [44]. Similarly, the *ITS/TEF1-α* which belongs to *L. theobromae* collected from the UAE showed 99% identity with that of both CMW 24701 and CMW 24702 strains isolated from *Eucalyptus* sp. in China [45]. None of the *ITS/TEF1-α* sequences that belong to *L. theobromae* including the pathogen from this study, clustered with other *Lasiodiplodia* spp. reported worldwide. This provides strong evidence that the isolate DSM 105134 in the current study belongs to *L. theobromae* sp. complex and is the main causal agent of dieback on mango in the UAE. Yet, it is probable this destructive strain of the fungal pathogen may have been introduced from Egypt.

This study was further extended to evaluate systemic fungicides to potentially control the pathogen under greenhouse and field conditions. There is now strong evidence that the indiscriminate use of chemicals does pose a potential risk to humans and other organisms, and unwelcomed side effects to the environment [46]. Yet, many studies urge to combine a number of antagonistic strategies, such as fungicides, biocontrol agents (BCA), and plant extracts, to prevent or reduce the activity of the pathogen growth and manage diseases in crops which, as a concept, is known as IDM [47,48]. IDM does not necessarily seek to eliminate the use of chemicals, but aims to minimize in a way that becomes least destructive to non-target life [49]. Several reports have focused on BCA against *L. theobromae*. In vitro studies showed that the antagonism of *Trichoderma* spp. (*T. harzianum* and *T. viride*) or *Aspergillus niger*, can be effective against *L. theobromae* [50,51]. Under laboratory and field conditions to protect bottle gourd (*Lagenaria siceraria*) against seedling and root rot diseases, plants treated with *Bacillus subtilis*, *T. harzianum* or *T. viride* were reported to reduce the pathogenic effect of *L. theobromae* by more than 90% [52]. Some researchers, on the other hand, have indicated that the efficacy of the BCA is dependent on many factors including the host age, the disease severity and the field environment. *Trichoderma* and other biological products, however, could serve as potential BCA against diseases associated with *L. theobromae*; indicating the potential for the integrated management of this disease. Until now, limited research has targeted the potential of the applicability of fungicides for the effective management of the dieback disease in mango.

With aim of searching for the successful fungicides to inhibit *L. theobromae*, we selected three systemic fungicides, Score^®^, Cidely^®^ Top, and Penthiopyrad^®^, and tested their efficacy under in vitro, greenhouse, and field conditions. All fungicides used in this study, in general, inhibited the fungus at the tested concentration (250 ppm), evidenced by the altered hyphal morphology, septum formation and the integrity of the cytoplasmic contents. Among all the fungicides tested, Cidely^®^ Top (difenoconazole and cyflufenamid) showed the strongest inhibition of mycelial growth with minor tolerance by the organism after 10 days of the in vitro experiment, and a significant reduction in disease symptoms in relation to the conidia counts in Cidely^®^ Top-treated seedlings at 2–4 wpt. This suggests that this fungicide may serve as a candidate fungicide for the management of *L. theobromae* affected mango trees. To a lesser extent, the difenoconazole-based fungicide, Score^®^, too was significantly effective in the reduction of the pathogenic activities of *L. theobromae* in both the laboratory and greenhouse trials. This result is in agreement with previous findings that this chemical does inhibit the growth of *L. theobromae* in vitro and in vivo [53,54], although higher concentrations of Score^®^ were used in their studies than was applied in our study. Although difenoconazole (Score^®^ and Cidely^®^ Top) was ineffective against *Fusarium magniferae* [55], this active ingredient was significantly effective and promising for managing other plant diseases [56,57,58], including dieback on mango (Figure 6). This could be attributed to the different growth conditions, fungicide application methods and/or the nature of responses to the chemicals by different fungal pathogens. It is noteworthy to mention that we found that the superior efficiency of the fungicide Cidely^®^ Top over Score^®^, may possibly be due to the additional presence of the active ingredient cyflufenamid, which may have contributed to the increased levels of inhibition of *L. theobromae*. Penthiopyrad showed high inhibitory activity against a wide range of plant pathogens, including *Rhizoctonia solani*, *Botrytis cinerea*, *Fusarium oxysporum*, and *Leptosphaeria* spp. [58,59]. Our data showed that the application of the carboxamide-based fungicide, Penthiopyrad^®^, was significantly effective in the reduction the pathogen hyphal growth and the production of mature conidia, in addition to causing hyphal swellings and cytoplasmic coagulation of *L. theobromae* compared to the PDA and seedling controls. The result obtained for carboxamide (Penthiopyrad^®^) seems to be in disagreement with a previous finding reporting that this chemical showed strong inhibitory activity of spore germination in various plant pathogens compared with that of mycelial growth of *B. cinerea* [58]. This discordance could be due to the preventive control effect of Penthiopyrad^®^ and the dosage of the fungicide treatment. To date, no reports exist relating to the evaluation of Cidely^®^ Top on mango trees infected with *L. theobromae*; while the same fungicide, however, was found to be highly effective against the pathogenic fungus *Thielaviopsis punctulata* on date palm [48]. Therefore, a field experiment was conducted to test the efficacy of Cidely^®^ Top, in infested mango orchards. Mango trees showed almost complete recovery, evident in the reduction of DSI by 54–91% in mango-sprayed trees with Cidely^®^ Top after 4 and 12 wpt compared to the untreated control. In conclusion, Cidely^®^ Top was useful in managing this destructive disease of mango in field, and could potentially be used as an effective component of IDM of dieback disease on mango.

″Omics″ are useful approaches to identify molecular changes that occur during disease or even prior to it, when prospective data are available [60]. Such data assume that the differences between healthy and diseased groups are directly related to disease [61,62]. This report focusing on the phenotype, i.e., symptoms associated with dieback disease, could be considered as a starting point for future comparative ″omic″ analyses including genomes and responses to environmental variation. Ultimately, we aim to reach towards full protection, which could ideally be achieved by the employment of IDM programs as well as ″omic″ approaches. In this research, we identified *L. theobromae*, for the first time, as the causal agent of dieback disease on mango in the UAE. We were also successful in finding a chemical means (viz. Cidely^®^ Top) to inhibit *L. theobromae* growth on mango trees. Investigation searching for other practices including IDM treatments to manage dieback in mango is in progress, ideally to promote the crop productivity and sustainability.

## 4. Materials and Methods

### 4.1. Fungal Isolation and Purification

Diseased trees in the Kuwaitat area in Al Ain City (Eastern region of Abu Dhabi Emirate; latitude/longitude: 24.21/55.74) with drying leaves on branches and twigs (Figure 1A–C) were studied in this investigation. A symptomatic tree (approximately five years old) was lifted and transferred to the Plant Microbiology Laboratory, Department of Biology, United Arab Emirates University in Al Ain City, for investigation. Longitudinal cross-sections were made of the diseased tree twigs and the pathogen was isolated from affected tissues (Figure S1). Tissues were cut into small pieces (2–5 mm long), washed, and surface-sterilized with mercuric chloride 0.1% for 1 min, followed by three consecutive washings in sterile distilled water. They were then transferred onto PDA (Lab M Limited, Lancashire, UK) plates, pH 6.0; supplemented with penicillin-streptomycin (Sigma-Aldrich Chemie GmbH, Taufkirchen, Germany) used at a rate of 25 mg/L of the growth medium in order to inhibit the bacterial contaminants. Petri dishes were incubated in an incubator at 28 ± 2 °C for five days. After this period of incubation, the mycelia growing out of the plated tissue was aseptically sub-cultured on fresh PDA and lastly purified by using hyphal-tip isolation technique [63]. The mycelium and conidia were observed using Nikon-Eclipse 50i light microscope (Nikon Instruments Inc., NY, USA) to characterize different fungal structures. A culture of the identified fungus, *L. theobromae* (Pat.) Griffon and Maubl. [13,14], has been deposited in Leibniz-Institute DSMZ- Deutsche Sammlung von Mikroorganismen und Zellkulturen GmbH (Braunschweig, Germany) under the collection number DSM 105134.

### 4.2. DNA Isolation, PCR, and Sequencing

The pathogen’s DNA from infected tissues of leaf, twig and apical shoot tips was extracted from mycelium cultured for 10–14 days at 28 °C on PDA plates, using the plant/fungi DNA isolation kit (Norgen Biotek Corp., Thorold, ON, Canada) with some modifications. Target regions of internal transcribed spacer (*ITS*) of the nuclear rDNA for *L. theobromae* using ITS1 and ITS4 primers [33], and partial *TEF1-α* using EF1-728F and EF1-986R [64] were amplified using the PCR. All primer sequence sets can be found in Table S1. All protocols for amplification and sequencing were as described [33].

### 4.3. Phylogenetic Analysis

For the analysis of the phylogenetic placement of the fungal isolate the sequences of *ITS* rDNA and *TEF1-α* genes were used as a single gene set and a concatenated two-gene set, ITS/*TEF1-α*. The obtained *ITS* and *TEF1-α* sequences were deposited in GenBank (accession numbers MF114110 and MF097964, respectively) and were further combined for constructing the phylogenetic tree against the *Lasiodiplodia* species database managed by the National Centre for Biotechnology Information (NCBI; www.ncbi.nlm.nih.gov). The *ITS*/*TEF1-a* sequence of the isolate from the UAE was aligned with sequences retrieved from GenBank, representing isolates that belong to about 18 species of the genus *Lasiodiplodia* [12,33]. All sequences were compared and aligned and maximum likelihood analyses were performed for estimation of the phylogenetic tree [65]. Phylogenetic trees were constructed and validated with a statistical support of the branches with 100 bootstrap resamples. These belong to isolates are: *L. plurivora*, *L. gilanensis*, *L. iraniensis*, *L. mahajangana*, *L. theobromae*, *L. hormozganensis*, *L. citricola*, *L. parva*, *L. egyptiacae*, *L. pseudotheobromae*, *L. crassispora*, *L. rubropurpurea*, *L. venezuelensis*, *L. gonubiensis*, *L. margaritaceae*, *Diplodia mutila*, *D. corticola*, and *Phyllosticta capitalensis*.

### 4.4. Disease Assays and Pathogenicity Tests

Inoculated detached mango (cv Badami) leaves (*n* = 12) were surface-sterilized with 70% ethanol before spray-inoculation with 5 × 10^4^ spores/mL of 10-day old culture of *L. theobromae* spore suspension. Control leaves were sprayed with sterilized distilled water without pathogen using a Preval sprayer (Valve Corp., Yonkers, NY, USA). Inoculated leaves were kept in a growth chamber at 28 °C and 80% relative humidity (RH). Inoculated detached leaves were examined for disease symptoms after five days.

Detached mango fruits (*n* = 12) were also tested to determine the effect of *L. theobromae*. Healthy mango fruits (cv Badami) were purchased from local fresh markets in the UAE. Fruits were stored at 4 °C and used within two days of purchase. Fruits were washed with sterile distilled water to remove dust and then the fruits were surface-sterilized with 70% ethanol. After air-drying in a flow cabinet, the mangoes were wounded using a sterilized scalpel (2 mm diameter, five wounds per mango), as described previously [66]. On each fruit, three agar plugs (11 mm in diameter) containing mycelium of *L. theobromae* (placed colonized surface down) and two agar control plugs containing no pathogen were applied. Inoculated fruits were further kept in a humid growth chamber at 28 °C and 80% RH, and were examined for disease symptoms after five days.

Disease was also assayed on whole mango seedlings (cv Badami). Twelve-month-old mango seedlings were inoculated with agar plugs (8 mm in diameter) containing mycelium of *L. theobromae* at the growing tip region of the stem, where the area of inoculation was wrapped with parafilm, as previously described [43]. Before inoculation, we surface-sterilized apical tips with 70% ethanol, and introduced mechanical wounding with sterilized scalpels. Control seedlings were treated/inoculated with PDA discs without pathogen. All inoculated seedlings were further maintained in a greenhouse with a photoperiod extended to 15 h under fluorescent lights (160 W/mol·m^2^·s) at 28 °C, and were examined for disease symptoms at 1, 3, and 5 wpi.

To satisfy Koch’s postulates, pieces of inoculated leaf and fruit tissues were removed from sites showing disease symptoms at 5 dpi, surface sterilized as mentioned above and plated on PDA. Similarly, pieces of infected stems showing disease symptoms at 5 wpi were surface sterilized as mentioned above, plated and incubated at 28 °C and the subsequent growth was recorded.

### 4.5. Evaluation of Fungicides Against L. theobromae

The fungicide experiment was carried out as previously described [48,57]. These fungicides selected were Score 250 EC^®^ (Difenoconazole; Syngenta International AG, Basel, Switzerland), Cidely^®^ Top 125/15 DC (Difenoconazole and Cyflufenamid; Syngenta), and Penthiopyrad 20SC^®^ (Carboxamide; Mitsui Chemicals Agro Inc, Tokyo, Japan). Each fungicide was dissolved in water with final concentrations of 0, 25, 75, 125, 250, 500, and 1000 ppm, and was then introduced aseptically into sterilized PDA at room temperature (RT). Penicillin-streptomycin antibiotics were added to inhibit the bacterial growth. The homogenized mixtures were aseptically poured into sterile Petri dishes. To introduce the tested pathogen on the control (without fungicide) and treatment (with fungicide) medium, a sterile cork-borer (8 mm diameter) was used. Cultures were incubated at 28 °C for 10 days, and the percentage of the mycelial growth inhibition was measured according to:

% *M*i = (*M*c − *M*t)/*M*c × 100% (1)

where *M*i = the inhibition of the mycelial growth; *M*c = the colony diameter (in mm) of the control set; and *M*t = the colony diameter (in mm) of the target fungus on the medium with fungicide.

An in vivo evaluation of the fungicides was also carried out on one-year-old mango seedlings (cv Badami) under greenhouse conditions, as described above. Seedlings were previously inoculated with agar culture discs containing mycelium of *L. theobromae* at the apical tip as described above. Inoculated seedlings were further kept in the greenhouse at 28 °C for two weeks (until disease symptoms were evident). Plants were then either sprayed with the fungicide (250 ppm; treatment) or with sterilized distilled water (control). Fungal conidia counts and the number of falling leaves were recorded at 2 and 4 wpt, as previously described [48]. Basically, the method of conidia counts involves homogenizing of known weight of affected tissues in 5 mL of water and assessing the suspended material to estimate the number of conidia using haemocytometer (Agar Scientific Limited, Essex, UK). It should be noted that leaf drop symptoms and fungal conidia counts were used for monitoring disease progression in the greenhouse experiment.

The field trials were performed in an orchard located in Abu Al-Abyad Island (Northern region of Abu Dhabi city, UAE; Latitude/Longitude: 24.20/53.80). Cidely^®^ Top was the only systemic fungicide tested on twelve mango trees cv Sindhri (four years old). Each tree was chosen so as to be surrounded by untreated trees (*L. theobromae* naturally-infested control) that can serve as a reservoir for recontamination. Treated mango trees were completely sprayed with the fungicide at the recommended dose used previously (250 ppm). The DSI corresponding to disease symptoms or recovery were recorded for disease assessment for fully grown trees at 4 and 12 wpt, using a scale of 0–5: 0 = no apparent symptoms, 1 = 1–10% necrotic, dark brown area on leaves or defoliating leaves, 2 = 11–25%, 3 = 26–50%, 4 = 51–75%, and 5 = 76–100% [67]. Experiments were repeated twice in March 2015 and March 2016 with similar results.

### 4.6. Statistical Analysis

For pathogenicity tests on leaves, fruits and seedlings, 12 tissues or seedlings for each treatment were used. For the in vitro evaluation of fungicides against *L. theobromae*, six plates for each treatment were used. For the fungal conidia counts and falling leaves of in vivo evaluation of fungicides under greenhouse conditions, a minimum of six plants for each treatment was used. Data represent the mean ± SD. Analysis of variance (ANOVA) and Duncan’s multiple range test were performed to determine the statistical significance at *p* < 0.05. All experiments were independently repeated three times with similar results.

For the DSI of the fungicide treatment in the field trials against *L. theobromae*, two replicates were tested. Data (mean ± SD) from a minimum of 12 plants per replicate were performed. Statistical significance at *p* < 0.05 was determined by ANOVA and Duncan’s multiple range test. Similar results were obtained in each replicate. 

All statistical analyses were performed by using SAS Software version 9 (SAS Institute Inc., Cary, NC, USA).

## Figures and Tables

**Table 1:** Disease severity index (DSI) after the application of Cidely^®^ Top on naturally-infested mango trees cv. Sindhri in the field (*n* = 12).

Treatment	DSI ^1^	
	4 Wpt	12 Wpt
*Lt*	3.42 (b)	4.42 (b)
CT	1.58 (a)	0.42 (a)

^1^ DSI is on a scale of 5: 0 = no infection, 1 = 1–10%, 2 = 11–25%, 3 = 26–50%, 4 = 51–75%, and 5 = 76–100% damage necrotic, dark brown area or defoliation in leaves. Values with different letters are significantly different from each other at *p* < 0.05. *Lt*, naturally-infested trees with *L. theobromae* only; CT, naturally-infested trees with *L. theobromae* sprayed with Cidely^®^ Top; wpt, weeks post-treatment.

## Supplementary Materials

Supplementary materials can be found at www.mdpi.com/1422-0067/18/10/2086/s1.

## References

[1] Berardini N., Fezer R., Conrad J., Beifuss U., Carle R., Schieber A. (2005). Screening of mango (*Mangifera indica* L.) cultivars for their contents of flavonol O- and xanthone C-glycosides, anthocyanins, and pectin. J. Agric. Food Chem..

[2] Nelson S. (2008). Mango anthracnose (*Colletotrichum gloeosporioides*). Plant Dis..

[3] Prakash O. (2003). Compendium of Mango Diseases and Disorders.

[4] Ploetz R.C., Ploetz R.C. (2003). Diseases of mango. Diseases of Tropical Fruit Crops.

[5] Ploetz R.C. (2004). The major diseases of mango: Strategies and potential for sustainable management. Acta Hortic..

[6] Smith P.F., Scudder G.K. (1951). Some studies of mineral deficiency symptoms in mango. Proc. Florida State Hort. Soc..

[7] Ramos L.J., Lara S.P., McMillan R.T., Narayanan K.R. (1991). Tip die back of mango (*Mangifera indica*) caused by *Botryosphaeria ribis.*. Plant Dis..

[8] Slippers B., Johnson G.I., Crous P.W., Coutinho T.A., Wingfield B., Wingfield M.J. (2005). Phylogenetic and morphological re-evolution of the *Botryosphaeria* species causing diseases of *Mangifera indica*. Mycologia.

[9] Van Wyk M., Al-Adawi A.O., Wingfield B.D., Al-Subhi A.M., Deadman M.L., Wingfield M.J. (2005). DNA based characterization of *Ceratocystis fimbriata* isolates associated with mango decline in Oman. Australas. Plant Pathol..

[10] Sakalidis M.L., Ray J.D., Lanoiselet V., Hardy G.E.ST., Burgess T.I. (2011). Pathogenic Botryosphaeriacea associated with *Mangifera indica* in the Kimberley region of Western Australia. Eur. J. Plant Pathol..

[11] Abdollahzadeh J., Javadi A., Mohammadi G.E., Zare R., Phillips A.J.L. (2010). Phylogeny and morphology of four new species of *Lasiodiplodia* from Iran. Persoonia.

[12] Ismail A.M., Cirvilleri G., Polizzi G., Crous P.W., Groenewald J.Z., Lombard L. (2012). *Lasiodiplodia* species associated with dieback disease of mango (*Mangifera indica*) in Egypt. Australas. Plant Pathol..

[13] Zambettakis E.C. (1954). Recherches sur la systematique des “Sphaeropsidales-Phaeodidymae”. Bull. Trimest. Soc. Mycol. Fr..

[14] Sutton B.C. (1980). The Coelomycetes, Fungi Imperfecti with Pycnidia, Acervuli and Stromata.

[15] Sharma I.M., Raj H., Kaul J.L. (1994). Studies on postharvest diseases of mango and chemical control of stem end rot and anthracnose. Indian Phytopathol..

[16] Ploetz R.C., Benscher D., Vázquez A., Colls A., Nagel J., Schaffer B. (1996). A re-examination of mango decline in Florida. Plant Dis..

[17] Al Adawi A.O., Deadman M.L., Al Rawahi A.K., Khan A.J., Al Maqbali Y.M. (2003). *Diplodia theobromae* associated with sudden decline of mango in the Sultanate of Oman. Plant Pathol..

[18] Khanzada M.A., Lodhi A.M., Shahzad S. (2005). Chemical control of *Lasiodiplodia theobromae*, the causal agent of mango decline in Sindh. Pak. J. Bot..

[19] De Oliveira Costa V.S., Michereff S.J., Martins R.B., Gava C.A.T., Mizubuti E.S.G., Câmara M.P.S. (2010). Species of Botryosphaeriaceae associated on mango in Brazil. Eur. J. Plant Pathol..

[20] Hong S.K., Lee S.Y., Choi H.W., Lee Y.K., Joa J.H., Shim H. (2012). Occurrence of stem-end rot on mango fruits caused *by Lasiodiplodia theobromae* in Korea. Plant Pathol. J..

[21] Haggag W.M. (2010). Mango diseases in Egypt. Agric. Biol. J. N. Am..

[22] Khanzada M.A., Lodhi A.M., Shahzad S. (2004). Mango dieback and gummosis in Sindh, Pakistan caused by *Lasiodiplodia theobromae*. Plant Health Prog..

[23] Naqvi S.A.H., Perveen R., Malik M.T., Malik O., Umer U.D., Wazeer M.S., Rehman A., Majid T., Abbas Z. (2014). Characterization of symptoms severity on various mango cultivars to quick decline of mango in district Multan. Int. J. Biosci..

[24] Khanzada M.A., Lodhi A.M., Rajput A.Q., Syed R.N., Shahzad S. (2015). Response of different mango cultivars to mango decline pathogen, *Lasiodiplodia theobromae* Pat.. Int. J. Biol. Biotechnol..

[25] Naqvi S.A.H., Perveen R. (2015). Mango quick decline manifestation on various cultivars at plants of particular age in the vicinity of district Multan. Pak. J. Phytopathol..

[26] Kazmi M., Fateh F., Majeed K., Kashkhely A.M., Hussain I., Ahmad I., Jabeen A. (2005). Incidence and etiology of mango sudden death phenomenon in Pakistan. Pak. J. Phytopathol..

[27] Paolinelli-Alfonso M., Villalobos-Escobedo J.M., Rolshausen P., Herrera-Estrella A., Galindo-Sánchez C., López-Hernández J.F., Hernandez-Martinez R. (2016). Global transcriptional analysis suggests *Lasiodiplodia theobromae* pathogenicity factors involved in modulation of grapevine defensive response. BMC Genom..

[28] Alemu K. (2014). Dynamics and management of major postharvest fungal diseases of mango fruits. J. Biol. Agric. Healthc..

[29] Asrey R., Patel V.B., Barman K., Pal R.K. (2013). Pruning affects fruit yield and postharvest quality in mango (*Mangifera indica* L.) cv. Amrapali. Fruits.

[30] Garg N., Pathak O., Pathak R.K. Use of Cow Dung Paste for Controlling Gummosis and Die Back Diseases of Mango. Proceedings of the 43rd Annual Conference of Association of Microbiologists of India.

[31] Gupta V.P., Tewar S.K., Govidaiah, Bajpai A.K. (1999). Ultrastructure of mycoparasitisms of *Trichoderma*, *Gliocladium* and *Laetisaria* species on *Botryodiplodia theobromae*. J. Phytopathol..

[32] Bhuvaneswari V., Rao M.S. (2001). Evaluation of *Trichoderma viride* antagonistic to post harvest pathogens on mango. Indian Phytopathol..

[33] Alves A., Crous P.W., Correia A., Phillips A.J.L. (2008). Morphological and molecular data reveal cryptic speciation in *Lasiodiplodia theobromae*. Fungal Divers..

[34] O’Callaghan M. (2016). Microbial inoculation of seed for improved crop performance: Issues and opportunities. Appl. Microbiol. Biotechnol..

[35] Usman M., Fatima B., Muhammad M.J. (2001). Breeding in Mango. Int. J. Agric. Biol..

[36] Lauricella M., Emanuele S., Calvaruso G., Giuliano M., D’Anneo A. (2017). Multifaceted health benefits of *Mangifera indica* L. (Mango): The inestimable value of orchards recently planted in sicilian rural. Nutrients.

[37] Punithalingam E. (1980). Plant Diseases Attributed to Botryodiplodia theobromae Pat.

[38] Cruywagen E.M., Slippers B., Roux J., Wingfield M.J. (2017). Phylogenetic species recognition and hybridization in *Lasiodiplodia*: A case study on species from baobabs. Fungal Biol..

[39] Rodríguez-Gálvez E., Alves A. (2015). Identification and pathogenicity of *Lasiodiplodia theobromae* causing dieback of table grapes in Peru. Eur. J. Plant Pathol..

[40] Mbenoun M., Momo Zeutsa E.H., Samuels G., Nsouga Amougou F., Nyasse S. (2007). Dieback due to *Lasiodiplodia theobromae*, a new constraint to cocoa production in Cameroon. New Dis. Rep..

[41] Amusa N.A., Adegbite A.A., Muhammed S., Baiyewu R.A. (2003). Yam disease and its management in Nigeria. Afr. J. Biotechnol..

[42] Twumasi P., Ohene-Mensah G., Moses E. (2014). The rot fungus *Botryodiplodia theobromae* strains cross infect cocoa, mango, banana and yam with significant tissue damage and economic losses. Afr. J. Agric. Res..

[43] Rodríguez-Gálvez E., Guerrero P., Barradas C., Crous P.W., Alves A. (2017). Phylogeny and pathogenicity of *Lasiodiplodia* species associated with dieback of mango in Peru. Fungal Biol..

[44] Pavlic D., Slippers B., Coutinho T.A., Gryzenhout M., Wingfield M.J. (2004). *Lasiodiplodia gonubiensis* sp. nov., a new *Botryosphaeria* anamorph from native *Syzygium cordatum* in South Africa. Stud. Mycol..

[45] Chen S.F., Pavlic D., Roux J., Slippers B., Xie Y.J., Wingfield M.J., Zhou X.D. (2011). Characterization of Botryosphaeriaceae from plantation-grown *Eucalyptus* species in South China. Plant Pathol..

[46] Aktar M.W., Sengupta D., Chowdhury A. (2009). Impact of pesticides use in agriculture: Their benefits and hazards. Interdiscip. Toxicol..

[47] AbuQamar S.F., Moustafa K., Tran L.S. (2017). Mechanisms and strategies of plant defense against *Botrytis cinerea*. Crit. Rev. Biotechnol..

[48] Saeed E.E., Sham A., Salmin Z., Abdelmowla Y., Iratni R., El-Tarabily K.A., AbuQamar S.F. (2017). *Streptomyces globosus* UAE1, a potential effective biocontrol agent for black scorch disease in date palm plantations. Front. Microbiol..

[49] Razdan V., Sabitha M., Peshin R., Dhawan A.K. (2009). Integrated disease management: Concepts and practices. Integrated Pest Management: Innovation-Development Process.

[50] Golam Mortuza M., Ilag L.L. (1999). Potential for biocontrol of *Lasiodiplodia theobromae (Pat.)* Griff. & Maubl. in banana fruits by *Trichoderma* species. Biol. Control.

[51] Adeniyi D.O., Adedeji A.R., Oduwaye O.F., Kolawole O.O. (2013). Evaluation of Biocontrol agents against *Lasiodiplodia theobromae* causing inflorescence blight of cashew in Nigeria. IOSR J. Agric. Vet. Sci..

[52] Sultana N., Ghaffar A. (2010). Effect of fungicides and microbial antagonists in the control of *Lasiodiplodia theobromae*, the cause of seed rot, seedling and root infection of bottle gourd. Pak. J. Agric. Res..

[53] Syed R.N., Mansha N., Khaskheli M.A., Khanzada M.A., Lodhi A.M. (2014). Chemical control of stem end rot of mango caused by *Lasiodiplodia theobromae*. Pak. J. Phytopathol..

[54] Rehman A.U., Naqvi S., Latif M., Khan S., Malik M., Freed S. (2015). Emerging resistance against different fungicides in *Lasiodiplodia theobromae*, the cause of mango dieback in Pakistan. Arch. Biol. Sci..

[55] Iqbal Z., Pervez M.A., Ahmad S., Iftikhar Y., Yasin M., Nawaz A., Ghazanfar M.U., Dasti A.A., Saleem A. (2010). Determination of minimum inhibitory concentrations of fungicides against fungus *Fusarium mangiferae*. Pak. J. Bot..

[56] Khan S.H., Idrees M., Muhammad F., Mahmood A., Zaidi S.H. (2004). Incidence of shisham (*Dalbergia sissoo* Roxb.) decline and in vitro response of isolated fungus spp. to various fungicides. Int. J. Agric. Biol..

[57] Saeed E.E., Sham A., El-Tarabily K.A., Abu Elsamen F., Iratni R., AbuQamar S.F. (2016). Chemical control of dieback disease on date palm caused by the fungal pathogen, *Thielaviopsis punctulata*, in United Arab Emirates. Plant Dis..

[58] Yanase Y., Katsuta H., Tomiya K., Enomoto M., Sakamoto O. (2013). Development of a novel fungicide, penthiopyrad. J. Pestic. Sci..

[59] Sewell T.R., Moloney S., Ashworth M., Ritchie F., Mashanova A., Huang Y.J., Stotz H.U., Fitt B.D.L. (2016). Effects of a penthiopyrad and picoxystrobin fungicide mixture on phoma stem canker (*Leptosphaeria* spp.) on UK winter oilseed rape. Eur. J. Plant Pathol..

[60] AbuQamar S.F., Moustafa K., Tran L.S. (2016). ‘Omics’ and plant responses to *Botrytis cinerea*. Front. Plant Sci..

[61] Sham A., Moustafa K., Al-Shamisi S., Alyan S., Iratni R., AbuQamar S. (2017). Microarray analysis of Arabidopsis *WRKY33* mutants in response to the necrotrophic fungus *Botrytis cinerea*. PLoS ONE.

[62] Sham A., Al-Azzawi A., Al-Ameri S., Al-Mahmoud B., Awwad F., Al-Rawashdeh A., Iratni R., AbuQamar S.F. (2014). Transcriptome analysis reveals genes commonly induced by *Botrytis cinerea* infection, cold, drought and oxidative stresses in *Arabidopsis*. PLoS ONE.

[63] Kirsop B.E., Doyle A. (1991). Maintenance of Microorganisms and Cultured Cells, a Manual of Laboratory Methods.

[64] Carbone I., Kohn L.M. (1999). A method for designing primer sets for speciation studies in filamentous ascomycetes. Mycologia.

[65] Tamura K., Stecher G., Peterson D., Filipski A., Kumar S. (2013). MEGA6: Molecular evolutionary genetics analysis version 6.0. Mol. Biol. Evol..

[66] Rungjindamai N. (2016). Isolation and evaluation of biocontrol agents in controlling anthracnose disease of mango in Thailand. J. Plant Prot. Res..

[67] Amponsah N.T., Jones E., Ridgway H.J., Jaspers M.V. (2012). Evaluation of fungicides for the management of *Botryosphaeria* dieback diseases of grapevines. Pest. Manag. Sci..

