# Supplementary material for: Detection and Management of Mango Dieback Disease in the United Arab Emirates
Source: Int J Mol Sci. 2017 Oct 20;18(10):2086. doi: 10.3390/ijms18102086 (PMC5666768; doi:10.3390/ijms18102086)
Supplement: Supplementary file 1 [file ijms-18-02086-s001.pdf]

Supplementary Materials: Detection and Management of Mango Dieback Disease in the United Arab Emirates

Esam Eldin Saeed, Arjun Sham, Ayah AbuZarqa, Khawla A. Al Shurafa, Tahra S. Al Naqbi, Rabah Iratni, Khaled El-Tarabily and Synan F. AbuQamar

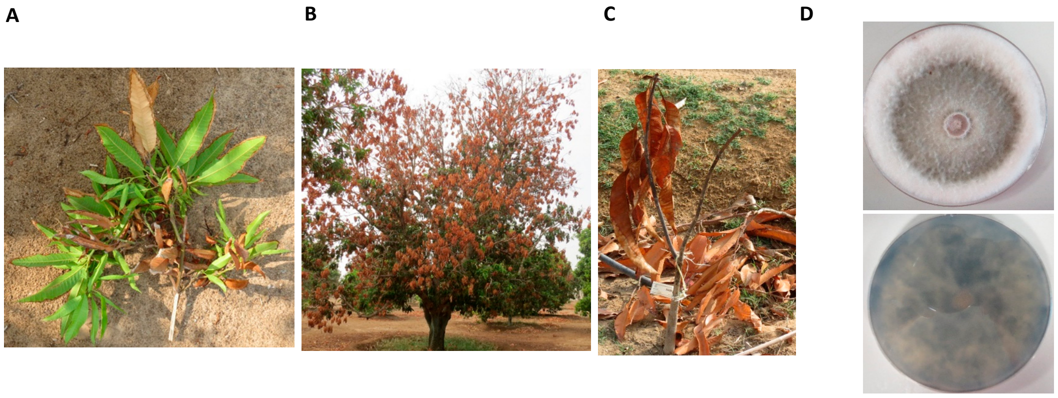

Figure S1. Additional symptoms of dieback disease on mango. Symptoms on (**A**) leaves; (**B**) whole tree; (**C**) disease affected small mango plant and (**D**) *L. theobromae* mycelia (top) with sporulation (bottom) on PDA plate. (**A**-**C**) naturally infested mango plants with *L. theobromae*; while (**D**) a 10-day old PDA culture.

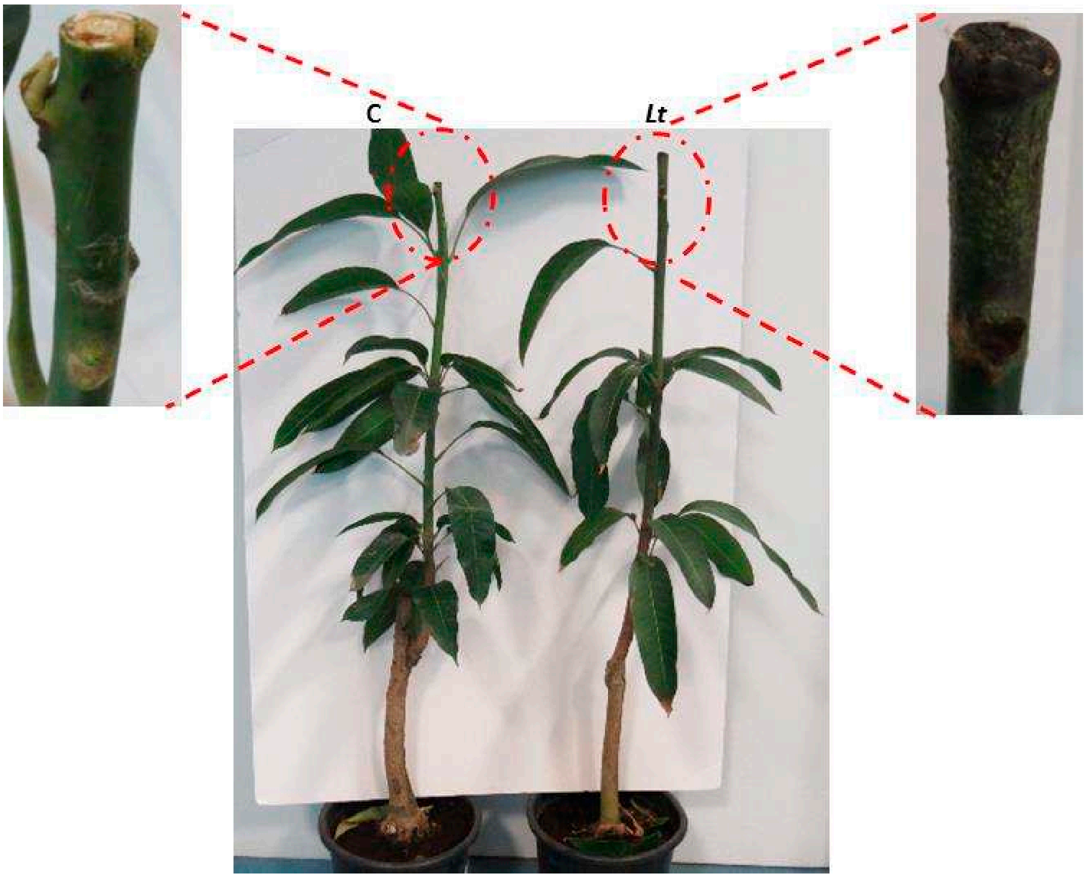

Figure S2. Pathogenicity test on seedlings inoculated with *L. theobromae*. *L. theobromae*-inoculated (*Lt*; right) and a non-inoculated control (C; left) seedling; and close-up photo of symptomatic tissues of the apical tip at 1 wpi. wpi, weeks post-inoculation.

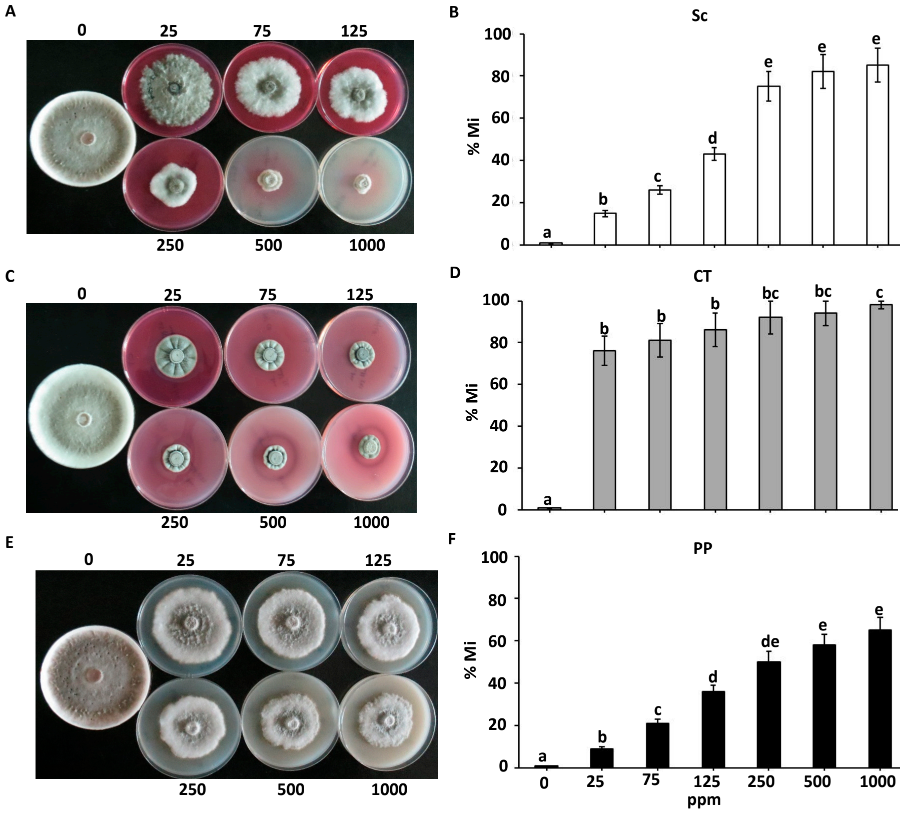

Figure S3. Growth inhibition effect of fungicides on *L. theobromae*. Growth inhibitory effect on *L. theobromae* using different concentrations (in ppm) of (**A**) Score®; (**C**) Cidely® Top; and (**E**) Penthiopyrad®. Growth inhibition rate (% Mi) of *L. theobromae* using different concentrations of (**B**) Score®, (**D**) Cidely® Top; and (**F**) Penthiopyrad®. Data were collected 10 days after inoculation. Values with different letters are significantly different at $p < 0.05$. Sc, Score®; CT, Cidely® Top; PP, Penthiopyrad®.

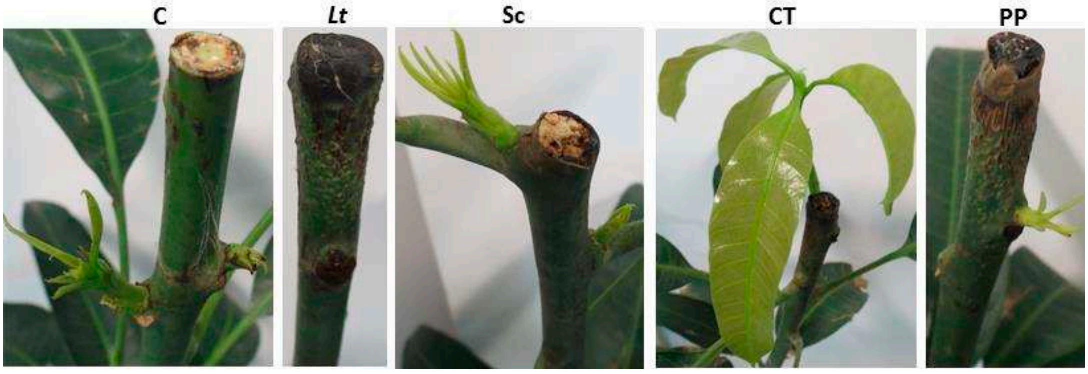

Figure S4. Impact of fungicides on the new growth of dieback diseased-mango seedlings. From left to right, non- inoculated control (C); inoculated with *L. theobromae* (*Lt*); inoculated with *L. theobromae* and sprayed with Score® (Sc); Cidely® Top (CT); Penthiopyrad® (PP) treatments at 4 wpt.

Table S1. List of PCR primers (sequence 5′ to 3′) used in this study.

Description	Left Primer Sequence	Right Primer Sequence
ITS	ITS1: TCCGTAGGTGAACCTGCGG	ITS4: TCCTCCGCTTATTGATATGC
TEF1-α	EF1-728F: TCATCGCAAGTCGAGAAGGT	EF1-986R: ACTTGAAGGAACCCTTACCG
ITS/LSU	*ITS/LSU*F: TACGCCGCATCCTTGCCGAG	*ITS/LSU*R: TTCCGTAGGTGAACCTGCGG
